# Association of HTR1F with Prognosis, Tumor Immune Microenvironment, and Drug Sensitivity in Cancer: A Multi-Omics Perspective

**DOI:** 10.3390/biomedicines13092238

**Published:** 2025-09-11

**Authors:** Yanjun Gao, Ziyue Zhang, Dafu Ye, Qingqing Li, Yingmei Wen, Shaowen Ma, Bo Zheng, Lei Chen, Yi Yao

**Affiliations:** 1 Cancer Center, Renmin Hospital of Wuhan University, Wuhan 430060, China; gaoyanjun@whu.edu.cn (Y.G.); zhangzy2015@whu.edu.cn (Z.Z.); ydf138711345452025@163.com (D.Y.); qqli1130@whu.edu.cn (Q.L.); wenyingmei@whu.edu.cn (Y.W.); bozheng3841@163.com (B.Z.); chenleiwhu@whu.edu.cn (L.C.); 2 Hubei Provincial Research Center for Precision Medicine of Cancer, Wuhan 430200, China; 3 Ningbo T-MAXIMUM Biopharmaceuticals Co., Ltd., Ningbo 315336, China; mashwen@163.com

**Keywords:** HTR1F, lung squamous cell carcinoma, immune infiltration, prognosis, multi-omics, MAPK signaling

## Abstract

**Background:** *HTR1F* (5-Hydroxytryptamine Receptor 1F) encodes a G protein-coupled receptor involved in serotonin signaling. Although dysregulated *HTR1F* expression has been implicated in certain malignancies, its biological functions and clinical significance across cancer types remain largely unexplored. **Methods:** We performed an integrative pan-cancer analysis of transcriptomic and pharmacogenomic datasets covering 34 cancer types (PAN-CAN cohort, N = 19,131; normal tissues, G = 60,499). Drug sensitivity and molecular docking analyses were conducted using the GSCALite database. The protein–protein interaction (PPI) network of *HTR1F* was constructed via the STRING database. Additionally, we evaluated the effects of *HTR1F* overexpression on proliferation and invasion in human lung squamous cell carcinoma (LUSC) cell lines NCI-H520 and NCI-H226. **Results:** *HTR1F* expression was significantly upregulated in 17 cancer types and was associated with poor prognosis, with LUSC showing an AUC of 0.912 for 1-year survival prediction. In LUSC, 695 genes were upregulated and 67 downregulated in response to *HTR1F* overexpression. *HTR1F* expression correlated with immune-related genes, immune checkpoints, tumor-infiltrating immune cells, tumor mutation burden (TMB), microsatellite instability (MSI), and drug responses. Genomic alterations, including amplification and deletion, were positively associated with *HTR1F* expression. Drug sensitivity analysis identified compounds such as sotrastaurin (−10.2 kcal/mol), austocystin D (−9.7 kcal/mol), and tivozanib (−9.3 kcal/mol) as potentially effective inhibitors based on predicted binding affinity. Functional enrichment analyses (GO, KEGG) and GSEA revealed that *HTR1F* is primarily involved in cell cycle regulation, DNA replication, cellular senescence, and immune-related pathways. Functional validation showed that *HTR1F* overexpression promotes proliferation of LUSC cells via the MAPK signaling pathway. **Conclusions:** Our integrative analysis highlights *HTR1F* as a potential biomarker associated with prognosis, immune modulation, and drug sensitivity across multiple cancer types. These findings provide a foundation for future experimental and clinical studies to explore *HTR1F*-targeted therapies.

## 1. Introduction

Cancer remains a major global cause of mortality [1,2]. In China and the USA, the new cancer cases estimated for the year 2022 are 4,820,000 and 2,370,000, respectively, with corresponding cancer-related deaths of 3.21 million and 640,000 [2].

Serotonin (5HT) or 5-hydroxytryptamine (5-HT) is a chemical messenger that regulates the neural activity and various vital neuropsychological processes [3,4]. Approximately 40% of the approved medicines target 14 different subtypes of 5-HT receptors, including a ligand-gated cationic pathway and 13 G-protein-coupled receptors (GPCRs) [5]. The 5-HT receptors are categorized into seven different 5-HT receptor (5HTR) families, among which HTR3, a ligand-gated ion channel, and most other identified HTRs are included in the category of G-protein-coupled receptors [6,7]. One of the seven 5HTR families is *5HTR1 (HTR1F)*, which includes *5-HT1A*, *5-HT1B*, *5-HT1D*, *5-HT1E*, and *5-HT1F* [8]. The messenger RNA (mRNA) of the *5-HT1F* receptor has been identified in the human brain, uterus, and mesenteric tissue, and also in vascular-related astrocytes, while it has not been detected in the heart, kidneys, liver, pancreas, spleen, or testicles so far [9,10]. The *5-HT1F* receptor is capable of coupling with Gi/o proteins, thereby attenuating adenylate cyclase activity, decreasing intracellular cAMP levels, and consequently reducing the phosphorylation of protein kinase A (PKA) and the activation of its downstream signaling pathways [11]. Lasmiditan, a drug approved for the treatment of acute migraine, activates the *5-HT1F* receptor in the CNS and blocks the release of CGRP and the neurotransmitter glutamate [11]. A positive expression of HTR1F in the mucous membrane tissues of colon cancer patients is reported to be significantly higher than that in the adjacent cancers. *HTR1F* is also associated with the development of bladder urinary epithelial carcinoma and may inhibit the transcriptional activity of the *erbB2* promoter, similar to the action of *HTR1E* [12].

Bioinformatics analyses of cancer-associated fibroblasts (CAFs) have identified ten CAF-related genes—*ANGPTL4*, *CPNE8*, *CST2*, *HTR1F*, *IL1RAP*, *NR1D1*, *NTAN1*, *OLFML2B*, *TMEM259*, and *VTN*—that exhibit strong prognostic significance for gastric cancer (GC) and provide substantial value in assessing patient survival outcomes [13]. Another bioinformatics analysis related to leukemia indicated that *HTR1F* could have a crucial role in *RUNX1* mutation acute myeloid leukemia (AML) [14]. Recent studies have revealed that serotonin (5-HT) plays complex and context-dependent roles in tumor immunity. Beyond its well-known functions in the nervous system, 5-HT can modulate the tumor immune microenvironment through multiple pathways. For example, evidence indicates that 5-HT can drive macrophage polarization toward a prolonged anti-inflammatory phenotype, primarily mediated by 5-HT2B and 5-HT7 receptors [15]. In contrast, T-cell lymphoma invasion and metastasis 2 (*TIAM2*) facilitates colorectal cancer development by sustaining a pro-inflammatory microenvironment through serotonin-dependent immunoregulatory pathways [16]. Furthermore, *5-HT1A* receptor signaling has been shown to create an immunosuppressive milieu in patients with lung adenocarcinoma and depression by activating p-STAT3 and autophagy pathways and upregulating downstream PD-L1 expression [17]. On the immune side, 5-HT1A receptors expressed on T cells promote the expansion of CD4(+)CD25(+)Foxp3(+) regulatory T cells while lowering the Th1/Th2 ratio, whereas on tumor cells, *5-HT1A* receptor activity negatively correlates with cytotoxic lymphocyte function. Additionally, blocking peripheral platelet-derived serotonin suppresses the growth of pancreatic and colorectal tumors in mice, enhances CD8(+) T-cell infiltration, and reduces PD-L1 levels within tumors [18]. These findings support the existence of a broader neuro-immune-tumor axis, in which multiple sources of 5-HT—including tumor cells, platelets, and infiltrating immune cells—may influence immune surveillance and tumor progression [19]. This framework provides an important biological context for evaluating the role of *HTR1F* in shaping immune cell infiltration. However, current evidence regarding the oncological roles of *HTR1F* is limited to a few cancer types, and its broader relevance across diverse tumor types remains unclear.

In this study, we conducted a comprehensive pan-cancer analysis of *HTR1F* using RNA-seq data from The Cancer Genome Atlas (TCGA), assessing its expression patterns, prognostic significance, and associations with tumor immune microenvironment characteristics such as immune cell infiltration, immune checkpoint expression, tumor mutation burden (TMB), and microsatellite instability (MSI). Functional enrichment analyses, including GO, KEGG, and GSEA, were employed to explore potential pathways. Furthermore, we performed drug sensitivity analysis and molecular docking to identify potential therapeutic agents targeting *HTR1F*. Finally, the biological function of *HTR1F* was validated in vitro using lung squamous cell carcinoma (LUSC) cell lines, where *HTR1F* overexpression was found to promote cell proliferation through activation of the *MAPK* signaling pathway. Together, our findings provide novel insights into the role of *HTR1F* in cancer progression and its potential as a therapeutic target.

## 2. Materials and Methods

### 2.1. Collection of Patients’ Data from Datasets

A uniformly processed pan-cancer dataset was obtained from the USC Xena platform (https://xenabrowser.net/), incorporating TCGA, TARGET, and GTEx data (PAN-CAN cohort, N = 19,131; G = 60,499). Expression profiles of the HTR1F gene were extracted for each sample, and cases were classified into the following categories: solid tissue normal, primary solid tumor, primary tumor, normal tumor, primary blood-derived cancer of bone marrow, and primary blood-derived cancer of peripheral blood. Cancer types represented by fewer than three samples were excluded, resulting in a final set of 34 tumor types with available expression data.

### 2.2. Differential Analysis and Prognostic Analysis

A log2 (x + 0.001) transformation was performed for each expression value of the transcriptome data from 34 tumors. Differential analysis and prognostic analysis of HTR1F in pan-cancer were performed via the “Gene expression prognostic analysis of pan-cancer analysis” module on a Chinese website (“http://www.sangerbox.com/home.html”). Differential expression analysis for *HTR1F* in LUSC was performed by dividing samples into high- and low-expression groups according to the mean *HTR1F* expression level. The analysis was carried out using the “limma” R package 3.64.3 [20], and genes with adjusted *p*-values < 0.05 and |fold change| > 1.5 were considered differentially expressed (DEGs).

### 2.3. Immune Cell Infiltration Analysis

Data were collected from the Tumor Immune Estimation Resource (TIMER, http://timer.comp-genomics.org/). Three different methods are available for predicting the immune cell infiltration level from the tumor transcription data—TIMER, CIBERSORT, and xCell. The correlation analysis of *HTR1F* with tumor immune cell infiltration in pan-cancer was performed using the “immune cell infiltration analysis of pan-cancer analysis” module on a Chinese website (“http://www.sangerbox.com/home.html”). Both websites are online platforms for data analysis and visualization.

### 2.4. Immunotherapy Prediction Analysis

Spearman correlation analyses between *HTR1F* expression and tumor mutational burden (TMB), microsatellite instability (MSI), as well as a panel of 60 immune checkpoint genes (including 24 inhibitory and 36 stimulatory genes), were conducted using the “Immune Checkpoint Blockade Analysis” module available on the Chinese bioinformatics platform SangerBox (http://www.sangerbox.com/home.html).

### 2.5. Functional Enrichment Analysis

To explore the biological functions and signaling pathways associated with HTR1F-related genes, Gene Ontology (GO) functional annotation and Kyoto Encyclopedia of Genes and Genomes (KEGG) pathway enrichment analyses were conducted using the R package clusterProfiler (v3.14.3). Terms with adjusted *p*-values < 0.05 were considered significantly enriched, and results were filtered based on enrichment scores to highlight the most relevant biological processes and pathways [21]. In parallel, Gene Set Enrichment Analysis (GSEA) was implemented through the dedicated GSEA R package to identify significantly enriched pathways at the transcriptome level.

### 2.6. Correlation Analysis Between HTR1F Expression and Clinical Factors

To comprehensively investigate the association between HTR1F expression and tumor progression across multiple cancer types, we employed the “Clinical Stage of Pan-Cancer Analysis” module provided by the SangerBox platform (http://www.sangerbox.com/home.html). This tool enabled us to examine the correlation between HTR1F levels and several clinical indicators, including tumor size and extent (T stage), overall pathological stage, and histological grade. Statistical comparisons between groups were conducted using an unpaired Student’s *t*-test for two-category variables and one-way ANOVA for multi-group analyses to determine significant expression differences among clinical stages. For lung squamous cell carcinoma (LUSC) specifically, the UALCAN web portal (http://ualcan.path.uab.edu/index.html) was used to generate visual representations of *HTR1F* expression across different clinical stages. The significance of expression differences was assessed via Student’s *t*-test, and results were annotated based on *p*-value thresholds: *p* < 0.05 (*), *p* < 0.01 (**), and *p* < 0.001 (***). Univariate and multivariate Cox regression and receiver operating characteristic (ROC) curve analyses were performed to evaluate the prognostic value of *HTR1F* in LUSC patients. The R package “survival” was employed to integrate the data of survival time, survival status, and clinical features and accordingly construct a nomogram using the Cox method to assess the prognostic significance of these features in LUSC patients.

### 2.7. Drug Sensitivity Analysis and Molecular Docking

The IC50 (half-maximal inhibitory concentration) and gene mRNA expression data of cancer cell lines were downloaded from GSCALite databases. The correlation between HTR1F expression level and drug sensitivity [half inhibitory concentration (IC50)] to 86 small molecule drugs in the GDSC database and CTRP database was analyzed.

In the molecular docking analysis, we utilized the PubChem database to identify the name, molecular weight, and 3D structure of small molecule drugs. The corresponding 3D structure of the *HTR1F* protein was retrieved from the RCSB PDB database (http://www.rcsb.org/). Molecular docking was conducted using CB-Dock2 website (https://cadd.labshare.cn/cb-dock2/php/index.php), where both ligands and proteins were prepared for docking.

The crystal structure of the *HTR1F* protein required preprocessing, including the removal of water molecules, hydrogenation, amino acid modifications, energy optimization, and adjustment of force field parameters to achieve the ligand’s low-energy conformation. Subsequently, the processed *HTR1F* protein structure was docked with the small molecule drug structures using the CB-Dock2 platform.

The docking results were evaluated based on the affinity (kcal/mol) value, which indicates the binding strength between the ligand and receptor. A lower affinity (kcal/mol) value signifies a more stable interaction between the ligand and the receptor.

### 2.8. Construction of the Protein–Protein Interaction (PPI) Network

An online tool for the retrieval of interacting genes (STRING 11.0; https://string-db.org/) was employed to predict the interactions of genes, both direct (physical) and indirect (functional) interactions, at the protein level [22]. The PPI network of DEGs was established using the criterion of high confidence >0.7. The PPI network was visualized in Cytoscape 3.8.0 software (https://cytoscape.org/) [23]. The hub genes were identified using the cytoHubba plug-in in the software [24].

### 2.9. Plasmid and Cell Lines

LUSC cell lines (NCI-H226 and NCI-H520) were obtained from the ATCC (American Type Culture Collection). The cell lines were cultured according to the provided instructions [25]. The Flag-HTR1F plasmid (Clone ID: OHu31121) was purchased from GenScript (Piscataway, NJ, USA). The HTR1F polyclonal antibody was purchased from Thermo Fisher (#PA5-106542, Waltham, MA, USA).

### 2.10. Cell Proliferation and Migration Function Assays

The CCK-8 (Cell Counting Kit-8) assay was employed to compare the cell viability between the negative control and the HTR1F high expression groups. The detailed procedures of the in vitro experiments are available in a previous report published by our research group [26]. The cell proliferation ability was assessed using the Cell Counting Kit-8 (CCK-8), CCK-8 assay kit was purchased from MedChemExpress (Monmouth Junction, NJ, USA). For this, 5 × 10^3^ cells per well were seeded into 96-well plates, with each condition performed in six replicates. The plates were incubated at 37 °C for 1 h, and the absorbance at 450 nm was measured daily over a span of 5 days.

To evaluate cell migration, transwell assays were performed using transwell chambers (Corning, NY, USA). Serum-free medium containing 1 × 10^5^ NIC-H226 or NIC-H520 cells was added to the upper chamber, while the lower chamber was filled with medium containing 10% FBS. After incubation at 37 °C for the designated period, cells that had traversed to the lower surface of the membrane were fixed and subsequently stained with Wright-Giemsa solution, which was purchased from Nanjing Jiancheng Bioengineering Institute (Nanjing, China). Migratory cells were quantified by counting in three randomly selected microscopic fields to obtain a representative estimate.

### 2.11. Western Blot

Total cellular proteins were isolated using RIPA lysis buffer supplemented with phosphatase and protease inhibitor cocktails (NCM Biotech, Shanghai, China) to prevent protein degradation and dephosphorylation. After SDS-PAGE separation, the denatured protein samples were electrophoretically transferred onto 0.4 μm polyvinylidene fluoride (PVDF) membranes at a constant current of 300 mA for 100 min. The membranes were then incubated with primary antibodies, followed by secondary antibodies. Protein signals were subsequently visualized using the ChemiDoc XRS+ system.

### 2.12. The Human Protein Atlas

The Human Protein Atlas (https://www.proteinatlas.org/) serves as a valuable online repository that integrates extensive transcriptomic and proteomic datasets derived from human tissues [27]. It covers expression profiles across 44 normal tissue types and 20 major tumor types. The platform also provides immunohistochemistry (IHC)-based protein expression data for both cancerous and corresponding normal tissues, which can be freely accessed and downloaded. In this study, the database was leveraged to explore the protein expression landscape of HTR1F in a variety of malignancies, offering insights into its potential role in tumorigenesis. Representative images were generated using antibody Sigma-Aldrich (St. Louis, MO, USA) Cat# HPA005555, RRID:AB_1856708.

### 2.13. Statistical Analysis

The resulting data were analyzed using the R software (version 3.6.4). A significant difference analysis was performed using the unpaired Student’s *t*-test and ANOVA to examine the differences among multiple groups of samples. The prognostic significance of variables was quantified by calculating hazard ratios (HRs) using both univariate and multivariate Cox regression models. To assess the association between the expression levels of two proteins, Pearson’s correlation analysis was conducted. Statistical significance levels were denoted as *p* < 0.05 (*), *p* < 0.01 (**), and *p* < 0.001 (***), and these annotations are consistently applied throughout all figures. Image data analysis was performed using ImageJ (version 1.54p). Furthermore, to evaluate the correlations between immune cell infiltration scores and gene expression across different tumor types, Pearson correlation coefficients were computed using the corr.test function from the psych R package (version 2.1.6), allowing identification of significantly associated immune infiltration parameters.

## 3. Results

### 3.1. HTR1F Expression and Survival Analysis in Pan-Cancer

**Figure 1:**
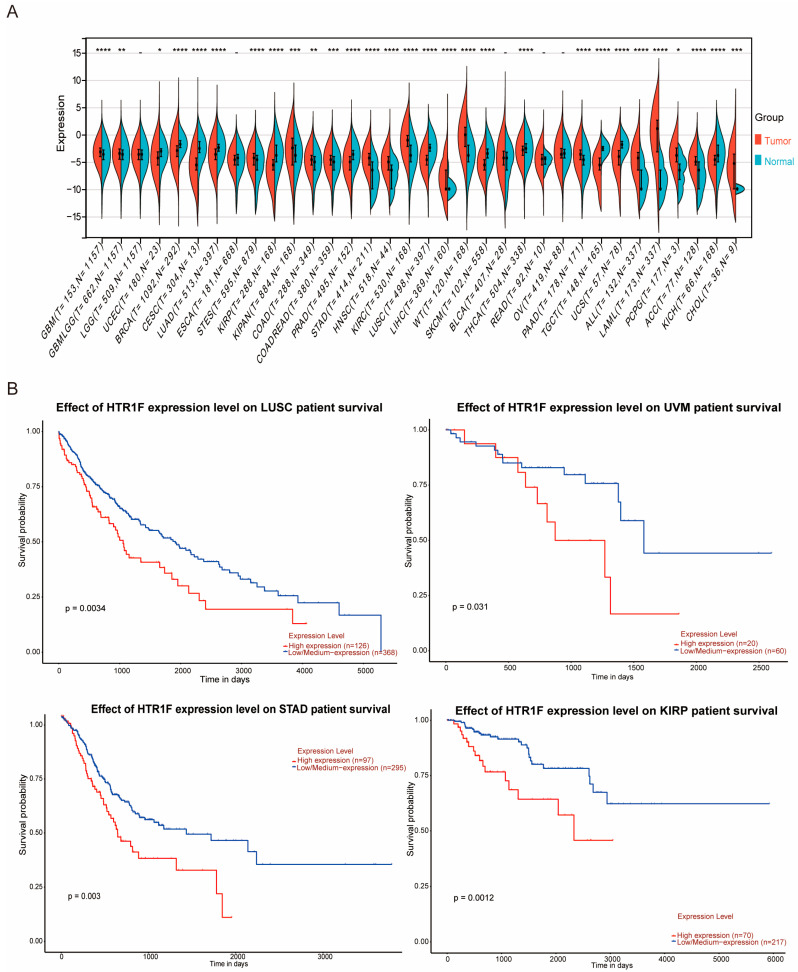
The elevated expression pattern of *HTR1F* in GC. (**A**) Differential expression of *HTR1F* between tumor and normal tissues in 34 cancer types from the TCGA and GTEx datasets. (**B**) The prognosis analysis of *HTR1F* gene expression in pan-cancer. (* *p* < 0.05; ** *p* < 0.01; *** *p* < 0.001; **** *p* < 0.0001).

The mRNA expression profile of *HTR1F* was assessed across multiple tumor types using RNA-seq data from 34 TCGA, TARGET, and GTEx cohorts (PANCAN, N = 19,131; G = 60,499) obtained from the UCSC database (Supplementary Table S1). Analysis demonstrated comparatively elevated *HTR1F* expression in 17 tumor types, including GBM, GBMLGG, STES, KIPAN, COAD, COADREAD, STAD, HNSC, KIRC, LIHC, WT, PAAD, ALL, LAML, PCPG, ACC, and CHOL (Figure 1A). Furthermore, the prognosis analysis of *HTR1F* gene expression in pan-cancer indicated a poor prognosis associated with the high expression of *HTR1F* in four tumor types—LUSC, KIRP, STAD, and UVM (Figure 1B). The differential analysis of *HTR1F* expression in pan-cancer revealed that *HTR1F* expression was significantly different in the gender subgroups of five tumor types (LGG, LAML, KIRP, KIPAN, and READ), histological grade in six tumor types (GBMLGG, LGG, ESCA, STES, STAD, and OV), and pathological stage in four tumor types (KIRP, KIPAN, THYM, and LIHC) (Supplemental Figure S1A–C). Representative immunohistochemistry images of HTR1F expression in normal and cancerous human tissues, including renal cancer, glioma, and stomach cancer, are shown from the Human Protein Atlas database (Supplementary Figure S2).

### 3.2. Tumor Immune Microenvironment Analysis of HTR1F Across Different Cancers

**Figure 2:**
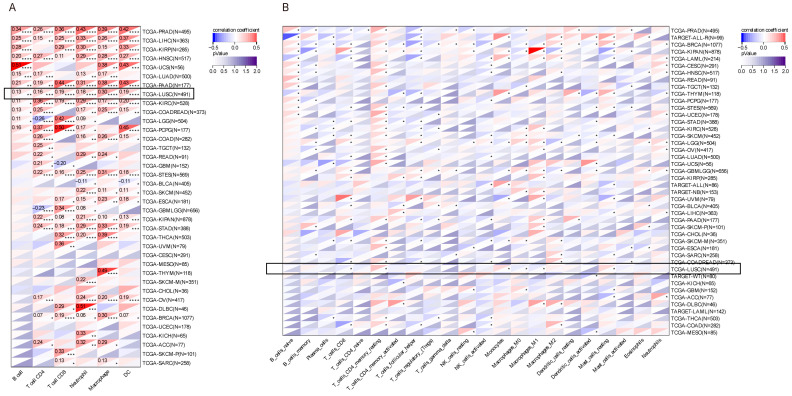
(**A**,**B**) Heatmaps illustrating the correlation between HTR1F expression and immune cell infiltration levels (X-axis) across 34 tumor types (Y-axis) (with a particular focus on LUSC, see black boxes). (**A**) TIMER and (**B**) CIBERSORT computational algorithms were used for immune cell quantification. * *p* < 0.05, ** *p* < 0.01, *** *p* < 0.001, **** *p* < 0.0001.

The tumor microenvironment (TME) constitutes a highly complex milieu comprising tumor cells, fibroblasts, immune cell populations, diverse signaling molecules, and the extracellular matrix (ECM) [28]. To explore the immunological relevance of *HTR1F* across cancers, its association with immune infiltration was systematically analyzed. Analysis using the TIMER database showed that *HTR1F* expression was significantly correlated with B cells, CD4^+^ T cells, CD8^+^ T cells, neutrophils, macrophages, and dendritic cells in multiple tumor types, especially PRAD, LIHC, KIRP, HNSC, UCS, LUSC, KIRC, COADREAD, LGG, and PCPG, with the correlation being particularly significant in LUSC (Figure 2A). Consistent findings were obtained using the CIBERSORT algorithm, which demonstrated that *HTR1F* expression is significantly related to 22 immune cell subsets across 42 commonly studied cancers (Figure 2B). In addition, the relationship between HTR1F expression and immune checkpoint regulators was evaluated, identifying 60 relevant genes—24 inhibitory and 36 stimulatory—that participate in immune checkpoint signaling pathways [29]. The results of the correlation analysis between *HTR1F* expression and the 60 immune checkpoint inhibitors indicated a significantly positive correlation of HTR1F expression with a majority of immune checkpoint inhibitors, especially in OV, STAD, UCS, LIHC, PRAD, HNSC, KIRC, and KIRP (Supplemental Figure S3A). Moreover, it was revealed that *HTR1F* was positively correlated with the majority of the immunomodulators, including chemokines, receptors, MHC, immuno-inhibitors, and immune stimulators in UVM, PAAD, PRAD, STAD, LIHC, OV, READ, KIRC, and KIRP (Supplemental Figure S3B). Nevertheless, it was noted that HTR1F expression had a significantly negative correlation with the tumor mutation burden (TMB) expression in four tumors (CESC, LAML, HNSC, and LUSC) (Supplemental Figure S4A). In addition, *HTR1F* expression presented a substantial correlation with microsatellite instability (MSI) in COADREAD, BRCA, STES, KIPAN, STAD, UCEC, and HNSC (Supplemental Figure S4B). The above results suggested that HTR1F could be potentially used for predicting the efficacy of ICIs (immune checkpoint inhibitors) in most cancers.

### 3.3. Genetic Alteration Analysis

**Figure 3:**
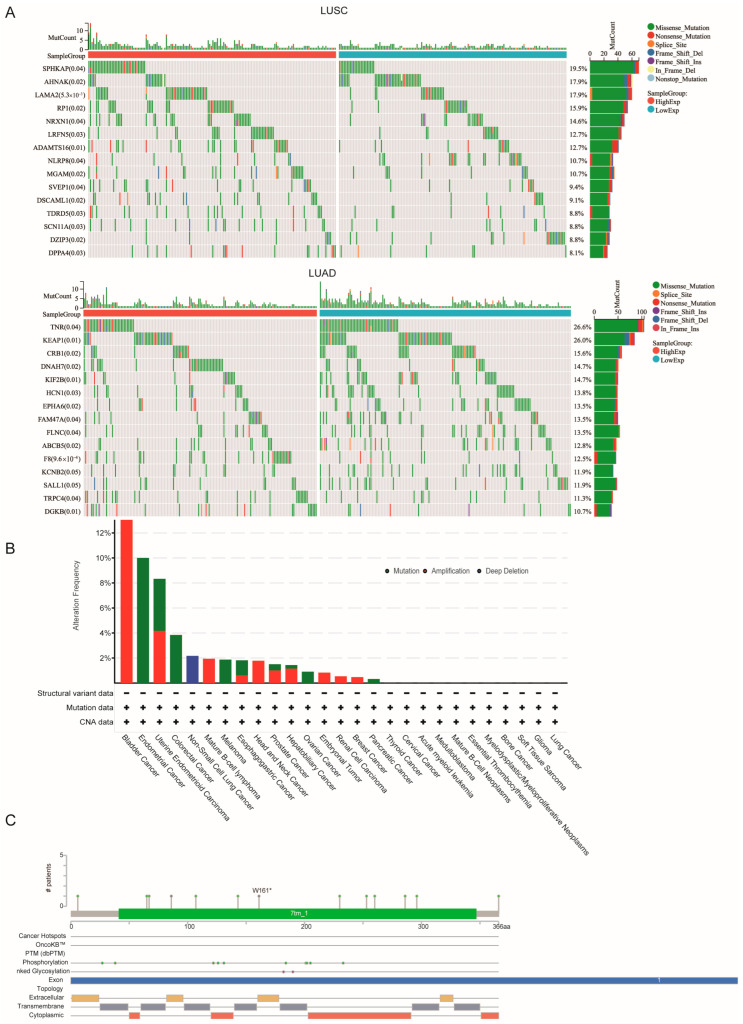
Mutation landscape of HTR1F. (**A**) The top five mutation genes between high and low expression of HTR1F in LUSC patients; (**B**) The top five mutation genes between high and low expression of HTR1F in LUAD patients; (**C**) Mutation landscapes of HTR1F for pan-cancer. * *p* < 0.05, ** *p* < 0.01, *** *p* < 0.001, **** *p* < 0.0001.

Tumor gene mutations are critical drivers of tumor growth and metastasis. This study focused on analyzing the mutation patterns of *HTR1F* across various tumor types. By comparing the mutation profiles between high-expression and low-expression groups of HTR1F, we identified several significantly mutated genes. In LUAD, notable mutations were found in TNR, KEAP1, and CRB1, while in LUSC, mutations were observed in SPHKAP, AHNAK, and LAMA2 (Figure 3A). We analyzed a range of tumor samples to investigate the genetic alteration landscape of *HTR1F*. As shown in Figure 3B, bladder cancer exhibited the highest alteration frequency of *HTR1F*, exceeding 12%, with mutations representing the most common alteration type. Additional information regarding the specific types, mutation sites, and the number of cases with *HTR1F* alterations is presented in Figure 3C.

### 3.4. Potential Chemo Drugs Targeting HTR1F in Pan-Cancer

**Figure 4:**
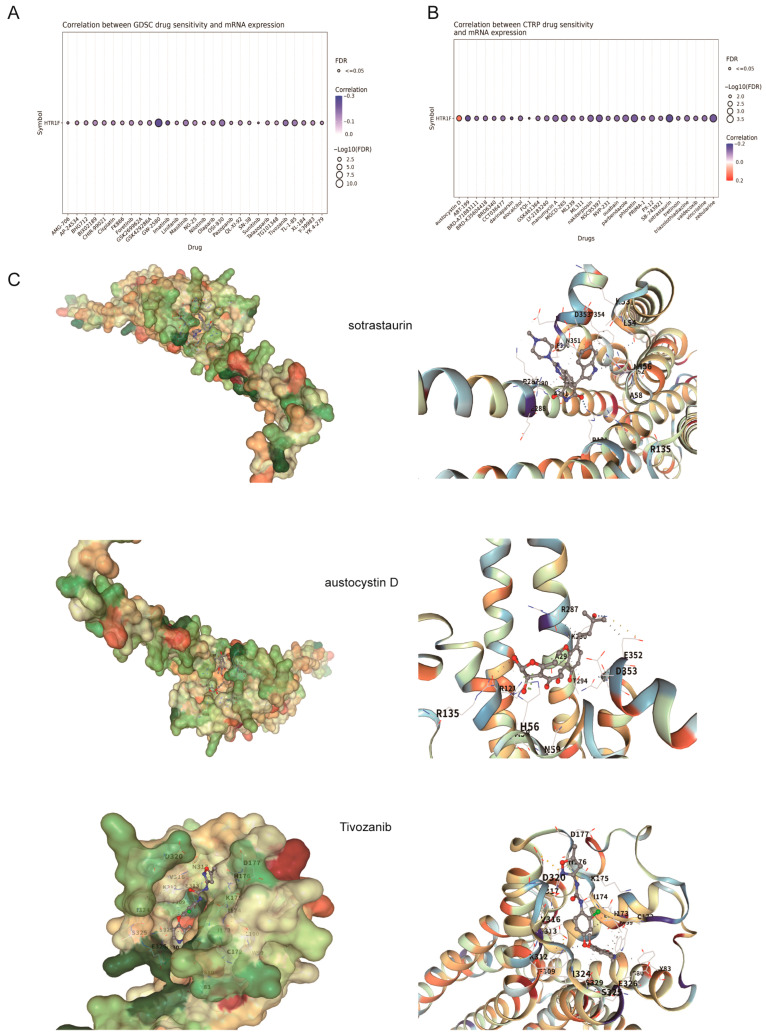
(**A**) Heatmap showing correlations between HTR1F expression and drug IC50 values from GDSC and (**B**) CTRP datasets; red indicates positive correlation, blue indicates negative. (**C**) Docking models of HTR1F with the top three drugs by binding affinity: sotrastaurin (−10.2 kcal/mol), austocystin D (−9.4 kcal/mol), and tivozanib (−9.0 kcal/mol).

Leveraging data from the GDSC and CTRP drug sensitivity databases accessed via the GSCALite online platform, the correlation between *HTR1F* gene expression and drug sensitivity was analyzed. The results revealed a significant positive association between high *HTR1F* expression and increased sensitivity to various small-molecule drugs, suggesting potential clinical applications for its elevated expression and related functions in LUSC (Figure 4A,B). Additionally, molecular docking simulations were conducted to evaluate the binding interactions between the *HTR1F* protein and the positively correlated drugs. As illustrated in Figure 4C, the three compounds exhibiting the strongest binding affinities were sotrastaurin (−10.2 kcal/mol), austocystin D (−9.4 kcal/mol), and tivozanib (−9.0 kcal/mol).

### 3.5. Clinical Correlation Analysis of HTR1F in Lung Squamous Cell Carcinoma

**Figure 5:**
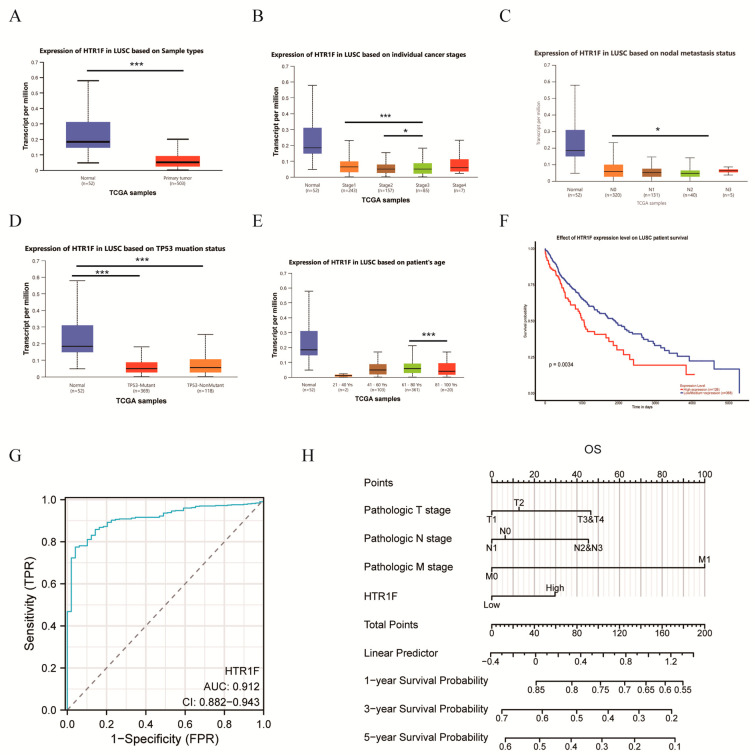
Clinical Correlation Analysis of *HTR1F* in LUSC. (**A**) The analysis of *HTR1F* expression between normal tissue and a primary tumor. Analysis of the clinical stage at cancer stage (**B**), nodal metastasis status (**C**), *TP53* mutation status (**D**), and age (**E**). (**F**) Survival curves for *HTR1F* expression in LUSC patients from the TCGA database. (**G**) Time-dependent ROC curve evaluating overall survival (OS) prediction based on *HTR1F* expression. (**H**) Nomogram estimating 1-, 3-, and 5-year OS probabilities in LUSC patients. Statistical significance is indicated as * *p* < 0.05, ** *p* < 0.01, *** *p* < 0.001, **** *p* < 0.0001.

Notably, HTR1F appeared to play a significant role in lung squamous cell carcinoma (LUSC), as illustrated in Figure 1 and Figure 2. To further clarify its clinical relevance, the relationship between HTR1F expression and clinicopathological features was examined using the UALCAN database. Elevated HTR1F expression was associated with primary tumor classification (Figure 5A), clinical stage (Figure 5B), nodal metastasis status (Figure 5C), TP53 mutation status (Figure 5D), and patient age (Figure 5E). The data obtained from the UALCAN database indicated that HTR1F overexpression was associated with poor prognosis in LUSC (Figure 5F). These findings suggested that HTR1F expression might be associated with malignant pathological progression in LUSC. Next, the prognostic efficacy of HTR1F in the overall survival rate of LUSC was evaluated based on ROC. The results indicated that the prognosis of HTR1F in LUSC could be suitably evaluated with the AUC values of 0.912 at 1 year (Figure 5G). In order to further evaluate the prognostic value of HTR1F in LUSC patients, a nomogram was constructed using HTR1F expression, pathological staging, and number of pack years smoked (Figure 5H). Altogether, it was demonstrated that HTR1F could serve as an independent prognostic indicator of LUSC in patients.

### 3.6. Potential Functional Analysis of HTR1F in LUSC Based on the TCGA Database

**Figure 6:**
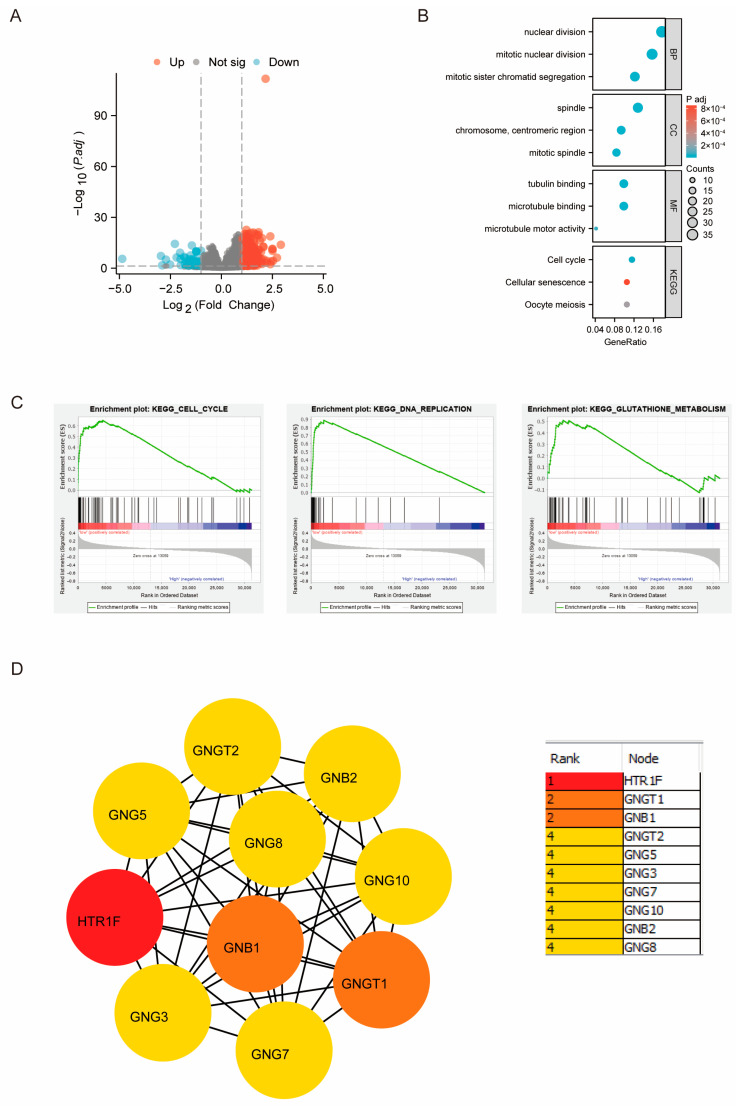
HTR1F-impacted signaling pathways in LUSC based on the TCGA database. (**A**) Volcano map for the differentially expressed proteins (*p* < 0.05, fold change > 1.5). (**B**) GO and KEGG analyses of HTR1F in LUSC. (**C**) GSEA analyses of HTR1F in LUSC. (**D**) Functional Protein Association PPI (Protein–Protein Interaction) network analysis using the Cytoscape software. The top nine genes are ranked by MCC score as GNGT1, GNB1, GNGT2, GNG5, GNG3, GNG7, GNG10, GNB2, and GNG8.

Using the thresholds of |fold change| > 1.5 and adjusted *p* < 0.05, 695 genes were identified as upregulated and 67 as downregulated in LUSC, as illustrated in the volcano plot (Figure 6A). To elucidate the biological functions associated with *HTR1F*, Gene Ontology (GO) and Kyoto Encyclopedia of Genes and Genomes (KEGG) enrichment analyses were conducted. GO analysis indicated that *HTR1F*-related genes were predominantly enriched in processes such as nuclear division, mitotic nuclear division, and mitotic sister chromatid segregation (Figure 6B). The KEGG enrichment analysis revealed that *HTR1F* was mainly involved in cell cycle, cellular senescence, and oocyte meiosis (Figure 6B). The GSEA also revealed that *HTR1F* is positively correlated with cell cycle, DNA replication, and glutathione metabolism (Figure 6C). In order to further evaluate the genes involved in *HTR1F* interaction, The PPI network statistics of *HTR1F*, which included 41 nodes (genes) and 303 edges (interactions), were obtained from the STRING database and then visualized using the Cytoscape software. The top nine hub genes were identified using the MCC method performed using the CytoHubba plug-in [19]. The nine hub genes were as follows: GNB1, GNGT1, GNG2, GNB3, GNB4, GNG13, GNG11, GNB5, and GNG4 (Figure 6D and Supplementary Table S1).

### 3.7. Effects of HTR1F on Cell Proliferation in LUSC

**Figure 7:**
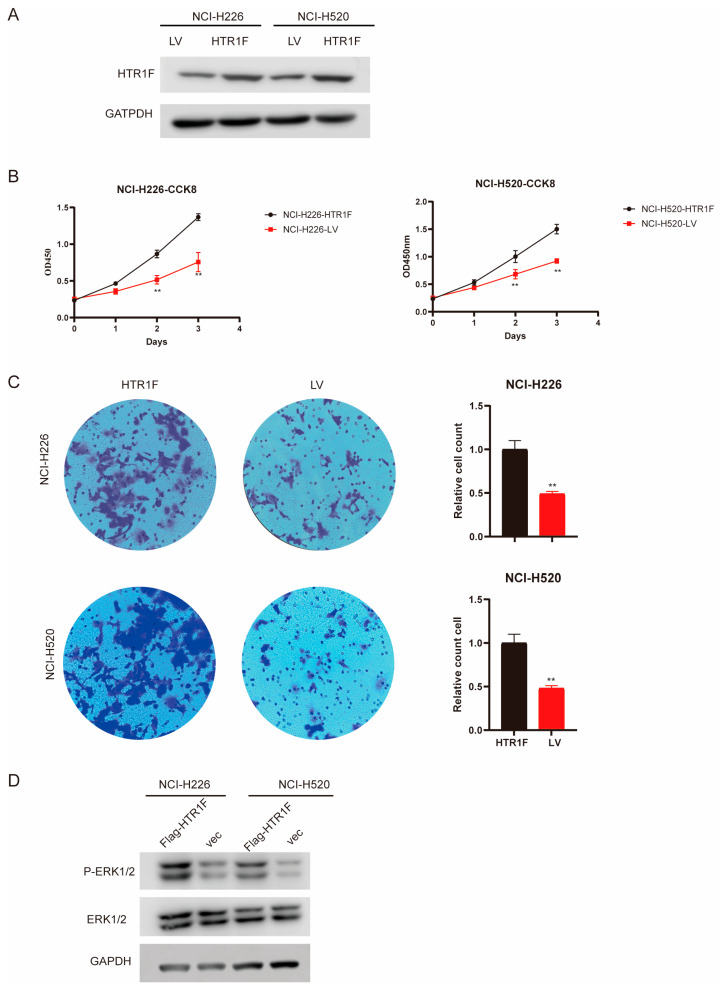
HTR1F inhibits the cell proliferation and metastasis of LUSC cell lines through the MAPK pathway. (**A**) Western blot assay confirmed the efficiency of HTR1F upregulation in NCI-H226 and NCI-H520 cells. (**B**) Cell proliferation was detected in NCI-H226 and NCI-H520 cells using the CCK8 assay. (**C**) Transwell migration assay images showing the number of migrated cells in NCI-H226 and NCI-H520 cells with HTR1F overexpression. (**D**) Western blot assay showed that HTR1F can regulate the MAPK signaling pathway. * *p* < 0.05, ** *p* < 0.01, *** *p* < 0.001, **** *p* < 0.0001.

Based on our previous findings, HTR1F was identified as an independent prognostic indicator for patients with lung squamous cell carcinoma (LUSC). However, its functional role in LUSC development required further experimental validation. To this end, we upregulated the expression of HTR1F in NCI-H226 and NCI-H520 LUSC cell lines. Western blot analysis confirmed successful upregulation of HTR1F protein levels in both cell lines (Figure 7A). Next, a CCK-8 assay was conducted to evaluate cell proliferation. The results showed that HTR1F overexpression significantly increased cell growth in both NCI-H226 (HTR1F vs. LV control, *p* < 0.01) and NCI-H520 cells (HTR1F vs. LV control, *p* < 0.01) (Figure 7B). In parallel, transwell assays revealed that HTR1F overexpression markedly promoted the invasive capacity of LUSC cells (Figure 7C). Mechanistically, Western blot analysis demonstrated that phosphorylated ERK1/2 (p-ERK1/2) levels were significantly induced upon HTR1F overexpression in both NCI-H226 and NCI-H520 cells, suggesting that HTR1F may increase LUSC progression via the MAPK signaling pathway (Figure 7D). These findings suggest that HTR1F overexpression is associated with enhanced proliferation and ERK activation; however, whether these changes depend on ligand-mediated receptor activation remains to be clarified.

## 4. Discussion

Previous studies have revealed that HTR1F is also associated with the development of bladder urinary epithelial carcinoma, GC prognosis, and the survival rate evaluation of the CAFs of GC [12,13]. However, systematic studies on the expression and roles of HTR1F in various types of tumors are warranted for further exploration in this research direction.

The results based on the TCGA database revealed that HTR1F was abnormally overexpressed in 17 types of tumors compared to the normal tissue and that HTR1F was associated with poor prognosis in four types of tumors. Next, the relationship between HTR1F and the clinical prognosis of LUSC patients was evaluated. It was revealed that the abnormal expression of HTR1F was closely linked to the prognosis and the clinicopathological stage of the patients with LUSC. A nomogram was constructed to evaluate the application of HTR1F in the prognostic assessment of LUSC. The above findings indicated that HTR1F can serve as a poor prognostic factor for tumors.

The tumor immune microenvironment is increasingly recognized as a decisive factor in determining whether tumor cells are eliminated by immune surveillance or evade immune control [30]. Consistent with previous studies demonstrating its close association with tumor progression, prognosis, and responsiveness to immunotherapy [31,32,33,34], our analysis using the TIMER database revealed that HTR1F expression is strongly correlated with the infiltration of diverse immune cell subsets—including B cells, CD4^+^ and CD8^+^ T cells, neutrophils, macrophages, and dendritic cells—across 34 cancer types. This widespread association suggests that HTR1F may play an important role in modulating the immune landscape of tumors, potentially influencing both tumor progression and therapeutic response. These findings highlight HTR1F as a candidate biomarker for tumor–immune interactions and warrant further investigation into its mechanistic contribution to immune regulation within the tumor microenvironment [35,36]. Our data further revealed that HTR1F expression significantly associates with increased infiltration of B cells, T cells, regulatory T cells (Tregs), and macrophages in various malignancies, underscoring its potential involvement in modulating the immune landscape. Given that immune checkpoint molecules have emerged as promising targets for cancer immunotherapy, the observed correlations suggest that HTR1F may be integrally linked to immune regulatory pathways within tumors, warranting further investigation into its role as a potential immunomodulatory factor and therapeutic target [36]. The present study also demonstrated that HTR1F was substantially related to several immune checkpoints and immunomodulators in various types of tumors. Tumor mutation burden (TMB) and microsatellite instability (MSI) reportedly affect the sensitivity of immunotherapy and the prognosis. Remarkably, in the present study, HTR1F expression exhibited a significantly negative correlation with tumor mutation burden (TMB) expression in four tumors (CESC, LAML, HNSC, and LUSC) and a positive correlation with the microsatellite instability (MSI) in COADREAD, BRCA, STES, KIPAN, STAD, UCEC, and HNSC. According to these findings, HTR1F could have an essential role in the regulation of the tumor microenvironment. Molecular docking simulations were conducted to evaluate the interactions between HTR1F protein and positively correlated drugs. Three drugs exhibiting the strongest binding affinities were sotrastaurin (−10.2 kcal/mol), austocystin D (−9.4 kcal/mol), and tivozanib (−9.0 kcal/mol). We emphasize that these analyses are exploratory and do not incorporate critical GPCR-specific features such as receptor conformational states, membrane environment, orthosteric constraints, or benchmarking against known 5-HT1F ligands. No experimental binding or functional pharmacology was performed. Therefore, the results should be interpreted as hypothesis-generating only and do not provide direct evidence of HTR1F engagement or therapeutic potential. To further investigate the molecular context of HTR1F, we constructed a protein–protein interaction (PPI) network to identify potential interacting partners. Gene Set Enrichment Analysis (GSEA)-based GO and KEGG enrichment analyses, using TCGA pan-cancer datasets, indicated that HTR1F is primarily involved in nuclear division–related biological processes and signaling pathways such as the cell cycle, cellular senescence, and oocyte meiosis. In addition, GSEA results demonstrated a positive association between HTR1F expression and pathways related to cell cycle regulation, DNA replication, and glutathione metabolism.

To validate the functional role of HTR1F in lung squamous cell carcinoma (LUSC), we performed HTR1F overexpression experiments in two LUSC cell lines, NCI-H226 and NCI-H520. The results showed that HTR1F upregulation significantly increased cell proliferation in both cell lines. However, the precise molecular mechanisms by which HTR1F exerts its regulatory effects in LUSC remain to be fully elucidated and warrant further investigation in future studies. In summary, our study revealed that HTR1F is markedly overexpressed in multiple tumor types, with its dysregulated expression closely linked to patient prognosis. Furthermore, aberrant HTR1F expression across pan-cancer was found to correlate significantly with the extent of immune cell infiltration, the expression levels of immune checkpoint molecules, and key genomic features such as tumor mutational burden (TMB) and microsatellite instability (MSI). These results suggest that HTR1F may play a critical role in shaping the tumor immune microenvironment and could serve as a valuable biomarker or therapeutic target in diverse cancers. Validation experiments confirmed that HTR1F has oncogene function and promotes the proliferation of lung squamous cell carcinoma (LUSC) through the MAPK pathway. Although HTR1F is a ligand-dependent GPCR, we did not assess ligand availability, receptor activation, or downstream signaling markers such as cAMP/β-arrestin responses or PTX sensitivity in this study. Therefore, the observed effects of HTR1F overexpression on cell proliferation and ERK phosphorylation should be interpreted as correlative rather than conclusive. Further studies will be required to determine whether these effects are strictly ligand-dependent. Therefore, it was concluded that HTR1F could be utilized as a potential prognostic biomarker and target for LUSC patients.

While this study represents the first comprehensive investigation into the role of HTR1F in cancer, several limitations should be acknowledged. First, our in vitro validation was limited to HTR1F overexpression in two LUSC cell lines (NCI-H226 and NCI-H520) without complementary loss-of-function or pharmacologic rescue experiments. Consequently, these findings are model-specific and preliminary, serving as proof-of-concept support for the pan-cancer bioinformatics analyses rather than definitive mechanistic evidence. Second, cross-cohort tumor–normal comparisons involve TCGA tumor and GTEx normal samples, which may be affected by batch effects due to differences in sample handling and experimental protocols; these comparisons should therefore be regarded as exploratory. Third, while the study incorporates some protein-level evidence from the Human Protein Atlas and Western blot validation in LUSC cell lines, the broader conclusions regarding HTR1F’s potential as a therapeutic target and clinical relevance are primarily based on transcriptomic data. Additional protein-level validation across diverse tumor types—using large-scale proteomic resources such as the Clinical Proteomic Tumor Analysis Consortium (CPTAC; https://proteomics.cancer.gov/programs/cptac, accessed on 10 August 2025)—would further strengthen these claims [37]. Fourth, our molecular docking results are intended solely to generate hypotheses rather than provide definitive structural or functional conclusions, and the ligand-dependence of HTR1F activity should be prioritized for direct biochemical or pharmacologic testing in future studies. Finally, as the transcriptomic and clinical data were obtained from multiple public datasets, the analyses may be influenced by systematic biases and dataset heterogeneity. Taken together, these limitations highlight the need for further experimental and clinical investigations to clarify the context-dependent functional roles of HTR1F across different cancer types.

## Data Availability

The datasets used or analyzed during the current study are available from the corresponding author on reasonable request.

## Supplementary Materials

The following supporting information can be downloaded at: https://www.mdpi.com/article/10.3390/biomedicines13092238/s1, Figure S1: Differential analysis of HTR1F expression with gender, histological grade, and pathological stage in pan-cancer; Figure S2: Representative immunohistochemistry (IHC) images showing HTR1F expression in normal and cancerous human tissues obtained from the Human Protein Atlas (HPA) database; Figure S3: HTR1F Correlations with Immune Checkpoints and Modulators in Cancer Immunity; Figure S4: The association of HTR1F with TMB and MSI in pan-cancer; Table S1: Top 10 Nodes in STRING (MCC).

## Abbreviations

TCGA-ACC	Adrenocortical carcinoma
TCGA-BLCA	Bladder urothelial carcinoma
TCGA-BRCA	Breast invasive carcinoma
TCGA-CESC	Cervical squamous cell carcinoma and endocervical adenocarcinoma
TCGA-CHOL	Cholangiocarcinoma
TCGA-COAD	Colon adenocarcinoma
TCGA-COADREAD	Colon adenocarcinoma/rectum adenocarcinoma esophageal carcinoma
TCGA-DLBC	Lymphoid neoplasm diffuse large B-cell lymphoma
TCGA-ESCA	Esophageal carcinoma
TCGA-GBM	Glioblastoma multiforme
TCGA-GBMLGG	Glioma
TCGA-HNSC	Head and neck squamous cell carcinoma
TCGA-KICH	Kidney chromophobe
TCGA-KIPAN	Pan-kidney cohort (KICH+KIRC+KIRP)
TCGA-KIRC	Kidney renal clear cell carcinoma
TCGA-KIRP	Kidney renal papillary cell carcinoma
TCGA-LAML	Acute myeloid leukemia
TCGA-LGG	Brain lower grade glioma
TCGA-LIHC	Liver hepatocellular carcinoma
TCGA-LUAD	Lung adenocarcinoma
TCGA-LUSC	Lung squamous cell carcinoma
TCGA-MESO	Mesothelioma
TCGA-OV	Ovarian serous cystadenocarcinoma
TCGA-PAAD	Pancreatic adenocarcinoma
TCGA-PCPG	Pheochromocytoma and paraganglioma
TCGA-PRAD	Prostate adenocarcinoma
TCGA-READ	Rectum adenocarcinoma
TCGA-SARC	Sarcoma
TCGA-STAD	Stomach adenocarcinoma
TCGA-SKCM	Skin cutaneous melanoma
TCGA-STES	Stomach and esophageal carcinoma
TCGA-TGCT	Testicular germ cell tumors
TCGA-THCA	Thyroid carcinoma
TCGA-THYM	Thymoma
TCGA-UCEC	Uterine corpus endometrial carcinoma
TCGA-UCS	Uterine carcinosarcoma
TCGA-UVM	Uveal melanoma
TARGET-OS	Osteosarcoma
TARGET-ALL	Acute lymphoblastic leukemia
TARGET-NB	Neuroblastoma
TARGET-WT	High-risk Wilms tumor
CI	Confidence interval.
GSEA	Gene set enrichment analysis
IC50	Half-maximal inhibitory concentration
DEGs	Differentially expressed genes
