# Supplementary material for: Association of HTR1F with Prognosis, Tumor Immune Microenvironment, and Drug Sensitivity in Cancer: A Multi-Omics Perspective
Source: Biomedicines. 2025 Sep 11;13(9):2238. doi: 10.3390/biomedicines13092238 (PMC12467612; doi:10.3390/biomedicines13092238)
Supplement: Supplementary file 1 [file biomedicines-13-02238-s001.zip › biomedicines-3794241-supplementary materials.pdf]

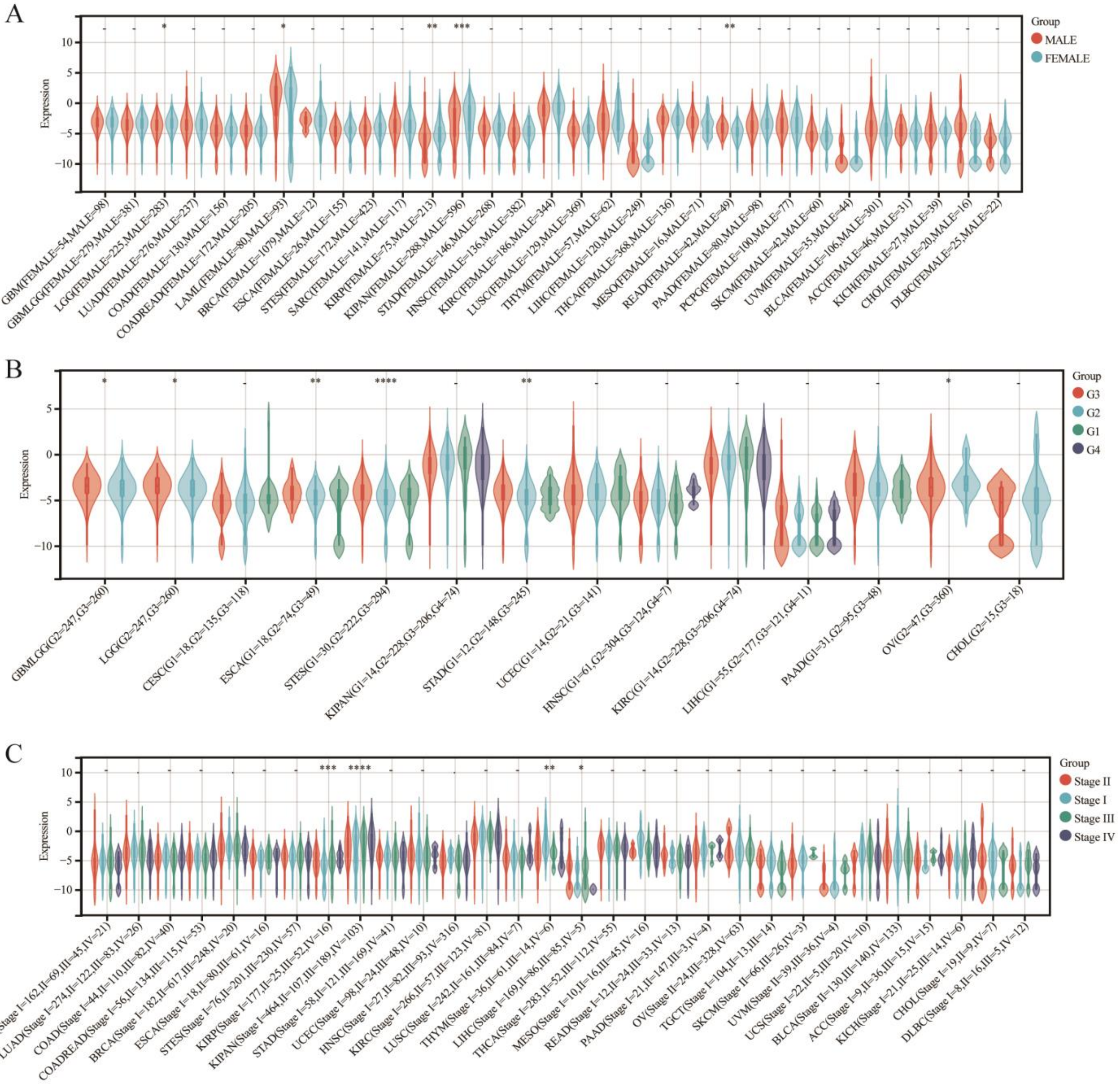

Supplementary Figure S1. Differential analysis of HTR1F expression with gender, histological grade, and pathological stage in pan-cancer. (A-C) (*$p < 0.05$; **$p < 0.01$; ***$p < 0.001$).

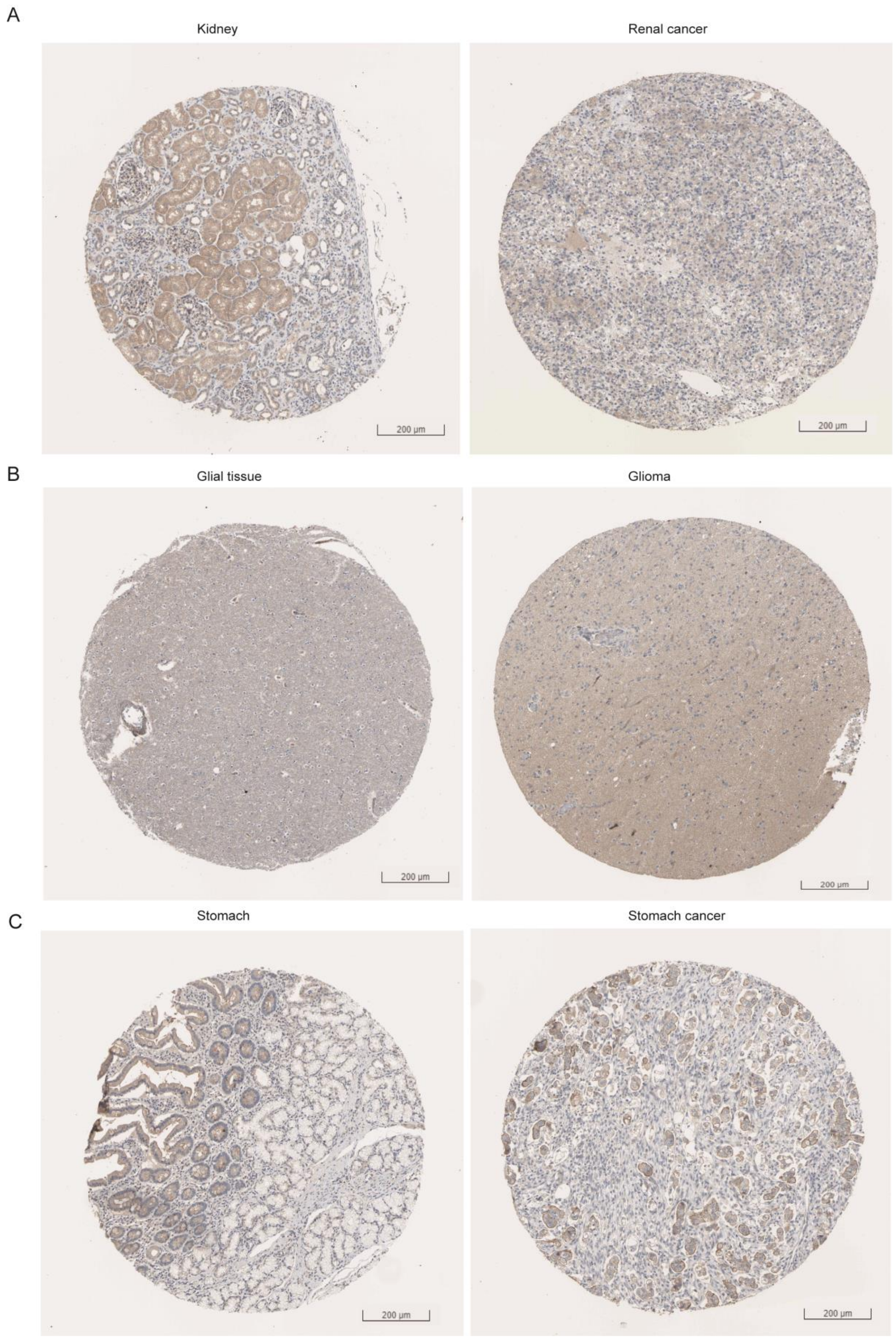

Supplementary Figure S2. Representative immunohistochemistry (IHC) images showing HTR1F expression in normal and cancerous human tissues obtained from the Human Protein Atlas (HPA) database. Staining was performed using antibody Sigma-Aldrich Cat# HPA005555,

RRID:AB_1856708.(**A**) Normal kidney (Patient ID: 2165) and renal cancer (Patient ID: 2540); (**B**) Normal glial tissue (Patient ID: 2523) and glioma (Patient ID: 1537);(**C**) Normal stomach tissue (Patient ID: 338) and stomach cancer (Patient ID: 2105). Scale bar = 200 μm. *$p < 0.05$; **$p < 0.01$; ***$p < 0.001$.

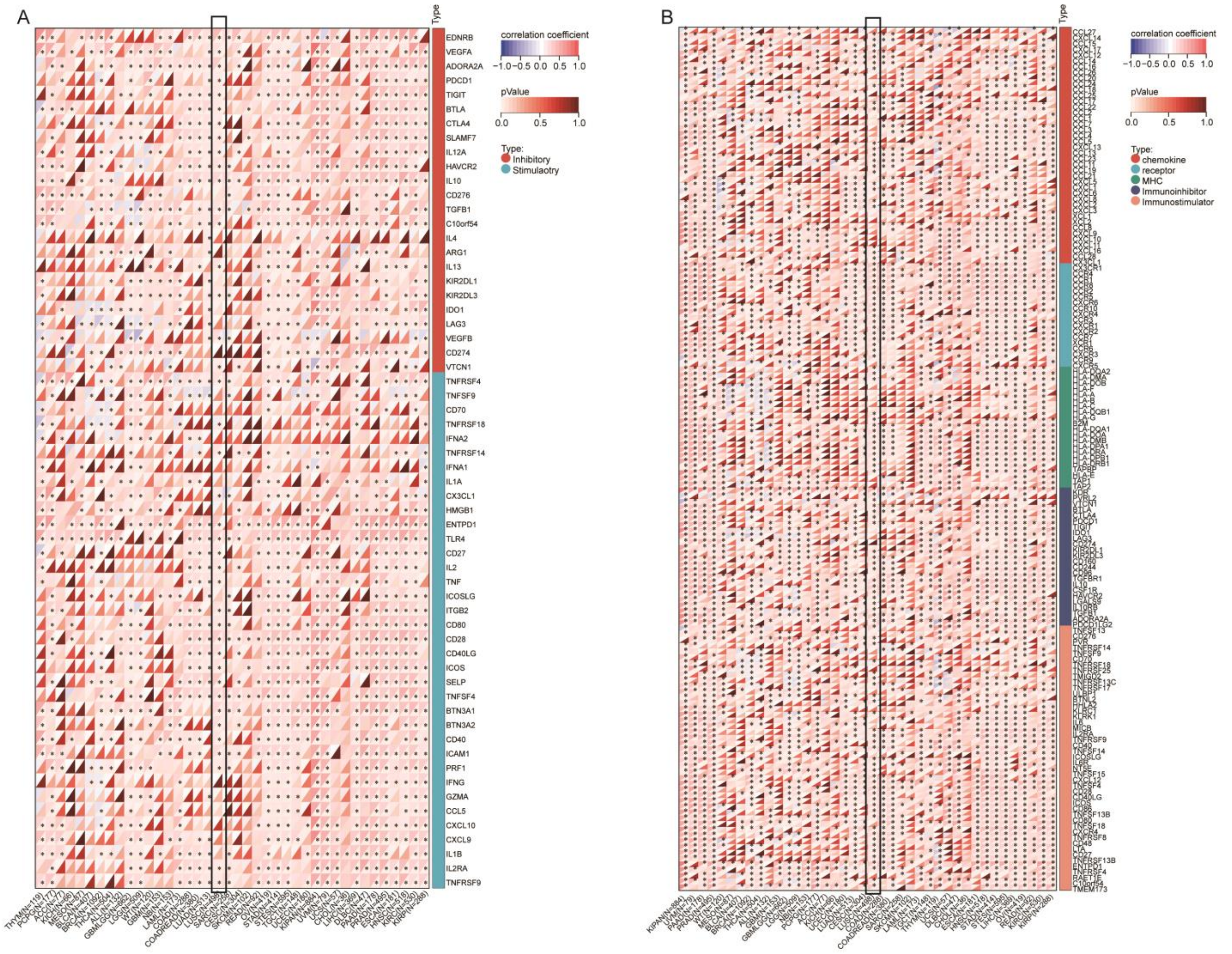

Supplementary Figure S3. HTR1F Correlations with Immune Checkpoints and Modulators in Cancer Immunity. (**A**) Analysis of correlations between HTR1F expression and immune checkpoint inhibitory genes(Y-axis) across diverse tumor types(X-axis) (with a particular focus on LUSC, see black boxes). (**B**) Correlation patterns between HTR1F and immune modulatory factors (Y-axis) among multiple cancers(X-axis) (with a particular focus on LUSC, see black boxes). Statistical significance is denoted by *$p < 0.05$, ** $p < 0.01$, and*** $p < 0.001$.

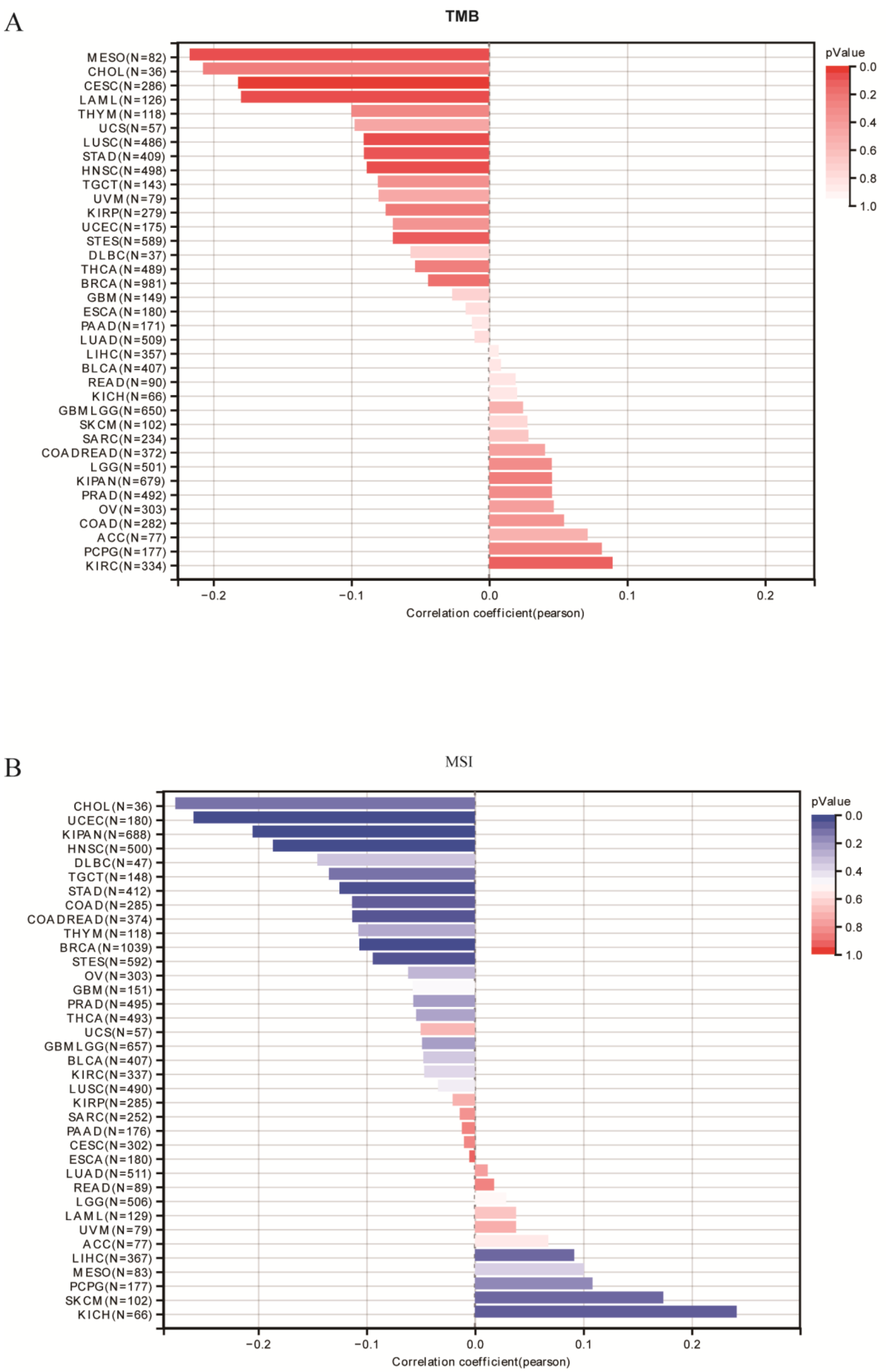

Supplementary Figure S4. The association of HTR1F with TMB and MSI in pan-cancer. (**A**) Correlation of the HTR1F expression levels with TMB. (**B**) Correlation of the HTR1F expression levels with MSI.
